# Impact of subanesthetic doses of ketamine on AMPA-mediated responses in rats: An *in vivo* electrophysiological study on monoaminergic and glutamatergic neurons

**DOI:** 10.1177/0269881115573809

**Published:** 2015-07

**Authors:** Kareem S El Iskandrani, Chris A Oosterhof, Mostafa El Mansari, Pierre Blier

**Affiliations:** University of Ottawa Institute of Mental Health Research, Mood Disorders Research, Ottawa, ON, Canada

**Keywords:** Ketamine, major depressive disorder, monoamines, glutamate, AMPA, NMDA

## Abstract

The rapid antidepressant action of a subanesthetic dose of ketamine in treatment-resistant patients represents the most striking recent breakthrough in the understanding of the antidepressant response. Evidence demonstrates tight interactions between the glutamatergic and monoaminergic systems. It is thus hypothesized that monoamine systems may play a role in the immediate/rapid effects of ketamine. *In vivo* electrophysiological recordings were carried in male rats following ketamine administration (10 and 25 mg/kg, i.p.) to first assess its effects on monoaminergic neuron firing. In a second series of experiments, the effects of ketamine administration on α-amino-3-hydroxy-5-methyl-4-isoxazolepropionic acid (AMPA)- and N-methyl-D-aspartate receptor (NMDA)-evoked responses in hippocampus CA3 pyramidal neurons were also investigated using micro-iontophoretic applications. Although acute (~2 hours) ketamine administration did not affect the mean firing activity of dorsal raphe serotonin and ventral tegmental area dopamine neurons, it did increase that of locus coeruleus norepinephrine neurons. In the latter brain region, while ketamine also enhanced bursting activity, it did increase population activity of dopamine neurons in the ventral tegmental area. These effects of ketamine were prevented by the prior administration of the AMPA receptor antagonist 2,3-dioxo-6-nitro-1,2,3,4-tetrahydrobenzo[*f*]quinoxaline-7-sulfonamide. An increase in AMPA-evoked response of CA3 pyramidal neurons was also observed 30 minutes following acute ketamine administration. The present findings suggest that acute ketamine administration produces a rapid enhancement of catecholaminergic neurons firing activity through an amplification of AMPA transmission. These effects may play a crucial role in the antidepressant effects of ketamine observed shortly following its infusion in depressed patients.

## Introduction

Several clinical studies have demonstrated the rapid but transient antidepressant effects of subanesthetic doses of the non-competitive NMDA receptor antagonist ketamine (Ki = 0.5 μM; Kapur and Seeman, 2002) in patients with treatment-resistant major depressive disorder (MDD). Significant improvement in depressive symptoms occurred within 72 hours following infusion of ketamine in seven subjects in the first placebo-controlled double-blind study (Berman et al., 2000). This was replicated in a larger double-blind study (Zarate et al., 2006). A larger two-site trial using the benzodiazepine midazolam to control for the psychotropic effects of ketamine confirmed a rapid onset of antidepressant effects (Murrough et al., 2013a). Moreover, repeated infusions of ketamine produced a more durable antidepressant response when compared to a single infusion (Murrough et al., 2013b).

Although studies have reported rapid antidepressant effects of ketamine 24 hours following an infusion, an immediate therapeutic effect of ketamine can manifest as early as an hour or two following intravenous infusions, once its psychotomimetic effects have subsided (Blier et al., 2012; Maeng and Zarate, 2007; Murrough et al., 2013b). The rapidity as well as the efficacy of the antidepressant response produced by ketamine in treatment-resistant patients is its most valuable asset. Understanding the mechanism behind these effects should bring the field a step closer to understanding and ultimately developing rapid, highly effective and mechanistically novel antidepressant treatments, leading to improved patient outcomes.

In preclinical studies, an increase in α-amino-3-hydroxy-5-methyl-4-isoxazolepropionic acid (AMPA) to N-methyl-D-aspartate receptor (NMDA) throughput is the predominant hypothesis posited to explain the rapid antidepressant effect of ketamine. Specifically, ketamine, by directly blocking NMDA receptors on gamma-aminobutyric acid (GABA) interneurons increases glutamate and leads to an increase in AMPA receptors activation (Abdallah et al., 2014; Homayoun and Moghaddam, 2007). It was also postulated that the sustained antidepressant effect of ketamine is likely achieved through an initiation of a number of downstream signaling pathways, including activation of the mammalian target of rapamycin (mTOR) pathway (Li et al., 2010, 2011) which results in an increase in translation of the brain-derived neurotrophic factor (BDNF) protein (Naughton et al., 2014; Zhou et al., 2013), ultimately leading to enhanced synaptic plasticity and neurotrophic changes (Li et al., 2011). However, changes in BDNF and mTOR expression and activation are only seen 24 hours following ketamine administration (Li et al., 2010). Further-more, while Li et al. (2010) showed that pre-administration of rapamycin blocked the ketamine-mediated decrease in immobility time in the forced swim test (FST), investigations by Autry et al. (2011) revealed that rapamycin failed to reverse such an effect. Hence, while enhanced neurotrophic factors might be involved in the sustained effects of ketamine observed 24 hours following its administration, the mechanisms behind the immediate effects remain elusive, suggesting that additional mechanisms might be involved.

All antidepressants currently in clinical use target one or more monoamine systems (El Mansari et al., 2010; Blier and El Mansari, 2013). Interactions between glutamate and monoamines are well documented and give rise to the interesting possibility that ketamine can produce, at least in part, its antidepressant effect by acting on monoamines neurotransmission (Paul and Skolnick, 2003; Millan 2006). The effect of NMDA receptor blockade on monoamines was already reported with phencyclidine (1-(1-phenylcyclohexyl)piperidine (PCP); Ki = 2 μM for NMDA receptor), a close congener of ketamine that is clinically not an antidepressant. Intravenous injection of PCP decreases firing rate of locus coeruleus (LC) norepinephrine (NE) neurons (Raja and Guyenet, 1980), while not modifying the firing activity of 5-HT neurons (Aghajanian et al., 1970; Raja and Guyenet, 1980). Another study demonstrated that intravenous ketamine increases firing of ventral tegmental area (VTA) dopamine (DA) neurons with potency 19 times weaker than the NMDA antagonist MK-801 (French and Ceci, 1990). Although some interactions between glutamate and monoamines have been documented, their exact functional connectivity remains to be investigated.

The present electrophysiological study was aimed at investigating whether a single dose of ketamine alters monoaminergic neuronal firing within a time frame whereby a therapeutic action can be observed, and whether any such changes are AMPA-dependent. Due to its pivotal role in neurocircuitry involved in major depression (see Drevets et al. 2008) and in light of studies showing neuronal plasticity following ketamine administration (see Kavalali and Monteggia, 2012), the hippocampus was selected to assess whether the responsiveness of CA3 pyramidal neurons to ketamine could as well promptly change following such a treatment. The results from this study could provide important insights in how the monoamine and glutamate systems interact, leading to better understanding of the mechanism by which ketamine produces its rapid antidepressant effect.

## Methods and materials

### Animals

The experiments were carried out in male Sprague–Dawley rats (Charles River, St Constant, Canada), weighing between 270 and 330 g at the time of the experiment. Rats were housed in groups of two per cage, under standard laboratory conditions (12:12 h light–dark cycle with access to food and water *ad libitum*). Body temperature was kept at 37°C during electrophysiological experiments. Animals were handled according to the guidelines of the Canadian Council on Animal Care (CCAC) and the local Animal Care Committee (Institute of Mental Health Research, Ottawa, Canada) approved protocols.

### Drug administration

Ketamine hydrochloride was dissolved in 0.9% aqueous saline solution. For acute experiments, ketamine was administered at a dose of 10 mg/kg and 25 mg/kg intraperitoneally (i.p.) at least 30 minutes prior to the electrophysiological experiments, as previously described (Li et al., 2010; Gigliucci et al., 2013). In the two-day administration paradigm, ketamine was administered at a dose of 10 mg/kg/day for two days, and an additional third injection was administered on day 3, at least 30 minutes prior to the electrophysiological experiments. Control rats on the other hand received the vehicle (0.9% aqueous saline solution). In the case where 10 mg/kg (Li et al., 2010) did not induce any effect, 25 mg/kg dose of ketamine was used as in Gigliucci et al. (2013); at this dose, ketamine is still subanesthetic and produces a significant effect in the FST model (Li et al., 2010; Gigliucci et al., 2013). The AMPA receptor antagonist 2,3-dioxo-6-nitro-1,2,3,4-tetrahydrobenzo[*f*]quinoxaline-7-sulfonamide (NBQX) was dissolved in 0.9% aqueous saline solution, and administered i.p. at a dose of 10 mg/kg for VTA experiments, and 3 mg/kg for LC experiments, 10 minutes prior to ketamine administration.

### In vivo electrophysiological experiments

Rats were anesthetized with chloral hydrate (400 mg/kg i.p) and mounted in a stereotaxic apparatus (David Kopf; Tujunga, CA, USA). Supplemental doses of the anesthetic (100 mg/kg, i.p.) were given to maintain constant anesthesia and prevent any nociceptive reaction to pinching of the hind paws. Extracellular recordings in the dorsal raphe nucleus (DRN) serotonin (5-HT), VTA DA and LC NE neurons were performed using single-barrel glass micropipettes (Stoelting, Wood Dale, IL, USA), pulled on a pipette puller (Narishige, Japan), and filled with 2 M NaCl solution and an impedance range of 2–4 MΩ. A burr hole was drilled at the stereotaxic coordinates corresponding to the brain structure of interest (Paxinos and Watson, 2007). The shape, duration of spikes, as well as the frequency of firing was used to identify neurons of interest, and recorded in real-time using the Spike2 program (Cambridge Electronic Design, Cambridge, UK).

For the recording of monoaminergic neurons, several electrode descents were made and neurons encountered were recorded in each brain structure to determine the effects of ketamine on the spontaneous firing rate of these neurons. Duration of recordings from the time of drug injection to the last neuron recorded varied between 100 and 140 minutes. Firing rate and percentage of burst firing were averaged to obtain the average firing rate and average burst activity of neurons in each rat.

### Recording of DRN 5-HT neurons

Electrodes were positioned 0.9–1.1 mm anterior to lambda on the midline and lowered into the DRN. Presumed DRN 5-HT neurons were encountered over a distance of 1 mm starting immediately below the ventral border of the Sylvius aqueduct. 5-HT neurons are then identified according to the following criteria: a slow (0.5–2.5 Hz), regular firing pattern, long duration and a positive action potential (VanderMaelen and Aghajanian, 1983). Investigations of the effects of ketamine on 5-HT neuron firing activity were conducted using two methods. In the first, firing of 5-HT neurons was assessed preceding and following ketamine administration in the same rat. The use of this method eliminated any observed variability that could occur between rats. In a second method, the firing activity of 5-HT neurons was assessed in rats that received either vehicle or ketamine.

### Recording of VTA DA neurons

Single-barrel glass micropipettes were positioned using the following coordinates (in mm from lambda): AP, +3.0 to +3.8; L, 1–0.6; V, 6.5–9. The presumed DA neurons were identified according to the well-established electrophysiological properties in vivo: a typical triphasic action potential with a marked negative deflection; a characteristic long duration (>2.5 ms) often with an inflection or ‘notch’ on the rising phase; a slow spontaneous firing rate (0.5–9 Hz) with an irregular single spiking pattern with burst activity (Ungless and Grace, 2012). The electrode was passed through the VTA in several tracks and spontaneously firing DA neurons were recorded. Population activity was determined as the number of neurons encountered in each rat divided by the number of tracks carried out (Grace and Bunney, 1983).

### Recording of LC NE neurons

Single-barrel glass micropipettes were positioned at 0.9–1.2 mm posterior to lambda and 0.9–1.3 to the midline suture. NE neurons were encountered at a depth of 5.5–7 mm from the surface of the brain. They are identified by their regular firing rate (0.5–5 Hz), a biphasic action potential of long duration (~2 ms), and a characteristic volley of spikes followed by a quiescent period in response to a nociceptive pinch of the contralateral hind paw (Cedarbaum and Aghajanian, 1977).

### Bursts analysis

The firing patterns of the monoaminergic neurons were analyzed by interspike interval (ISI) burst analysis. The onset of a burst was signified by the occurrence of two spikes with ISI < 0.08 s for NE and DA, and ISI < 0.01 s for 5-HT. The termination of a burst was defined as an ISI > 0.16 s for NE and DA (Dawe et al., 2001; Grace and Bunney, 1983) and ISI > 0.01 s for 5-HT (Hajos and Sharp, 1996).

### Microiontophoresis and extracellular recording of dorsal hippocampus CA3 pyramidal neurons

Extracellular recording and microiontophoresis of glutamatergic CA3 pyramidal neurons were carried out using five-barreled glass micropipettes with a tip broken back to 10–12 μm. The central barrel used for the unitary recording was filled with a 2 M NaCl solution, and the impedance of these electrodes ranged from 2 to 4 MΩ. The side barrels were filled with the following solutions: AMPA hydrobromide (5 mM in 200 mM NaCl, pH 8), NMDA (10 mM in 200 mM NaCl, pH 8) and 2 M NaCl solution for automatic current balancing. The micropipettes were lowered into the dorsal CA3 region of the hippocampus using the following coordinates: 4.0 mm anterior to lambda and 4.2 mm lateral. CA3 pyramidal neurons were found at a depth of 4.0 ± 0.5 mm below the surface of the brain. Since pyramidal neurons do not discharge spontaneously in chloral hydrate anesthetized rats, a small current of AMPA (-2 to -5 nA) was constantly applied to locate those neurons, and activate them within their physiological firing range (10–15 Hz; Rank, 1975). When AMPA and NMDA were not ejected, a retention current of +15 nA was applied to prevent leakage from the barrels. Pyramidal neurons were identified by their large amplitude (0.5–1.2 mV) and long-duration (0.8–1.2 ms) simple action potentials, alternating with complex spike discharges (Kandel and Spencer, 1961). The duration of microiontophoretic ejections of NMDA and AMPA was constant at 60 s. During these experiments, both the duration and current of NMDA and AMPA for microiontophoresis ejection remained the same before and after the i.p. injection of ketamine or saline. Drug effect was assessed by measuring the degree of excitation of pyramidal neurons (measured as number of spikes generated for 60 s ejection) induced by NMDA and AMPA applications following the acute administration of ketamine or saline. Results are expressed as overall changes in the percentage of baseline firing rate of dorsal hippocampus CA3 pyramidal neurons following administration of ketamine or vehicle.

### Statistical analysis

Data are expressed as means ± SEM. In DRN, LC and VTA, comparisons between controls and treated groups were carried out using one-way analysis of variance (ANOVA) followed by a Tukey post hoc test. Analysis of data from microiontophoresis was carried out using the two-way ANOVA with repeated measures and the Bonferroni post-hoc analysis was conducted when significant ANOVA results were obtained. These comparisons were statistically analyzed and graphed using the software Graphpad (Prism software Inc, La Jolla, CA). In all data analysis, statistical significance was taken as *p*<0.05. Burst activity of DA and NE neurons was analyzed with burstiDAtor software (www.github.com/nno/burstidator/releases).

### Drugs

Ketamine hydrochloride was purchased from ERFA Canada Inc. (Montreal, QC, Canada). AMPA hydrobromide, NMDA and NBQX were both purchased from Tocris Biosciences (Ellisville, MO, USA). Chloral hydrate was purchased from Sigma-Aldrich Canada Co. (Oakville, ON, Canada).

## Results

### Effects of acute and two-day administration of ketamine on the firing activity of DRN 5-HT neurons

**Figure 1.**
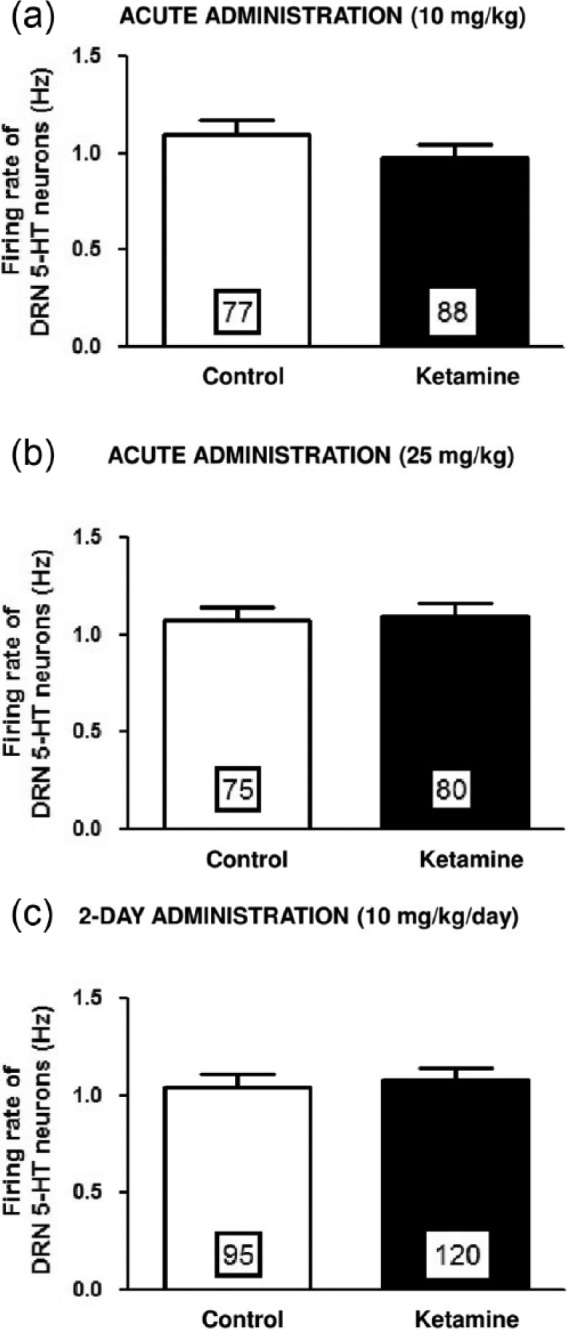
Effects of acute and two-day administration of ketamine on DRN 5-HT neuron firing. Mean (± SEM) of the firing rate of 5-HT neurons following acute (a) and (b) and two-day (c) administration of vehicle or ketamine at a dose of 10 mg/kg/day (a) and (c) and 25 mg/kg/day (b). Numbers in the histograms correspond to the number of neurons recorded (5–6 rats tested per group).

Acute administration of ketamine (10 mg/kg; i.p.) yielded no significant change in the average firing activity of 5-HT neurons in the DRN when the paradigm in which firing was assessed prior to and following ketamine administration in the same rat. Similarly there was no significant alteration in 5-HT neurons firing in the paradigm in which vehicle and ketamine administered rats were used (Figure 1(a)).

A previous study showed that 25 mg/kg but not 10 mg/kg of ketamine elicited and antidepressant-like effect in the FST model (Gigliucci et al., 2013; Li et al., 2010). Therefore, to rule out the possibility that an insufficient dose of ketamine was used, a higher dose of 25 mg/kg was tested, but still did not produce any alteration of the firing activity of 5-HT neurons (Figure 1(b)).

In addition, no significant change in the proportion of neurons exhibiting burst activity was observed with either dose (vehicle: 22%; ketamine 10 mg/kg: 28%, and 25 mg/kg: 33%). Hence the firing activity of 5-HT neurons remained unaltered following acute administration of ketamine.

A two-day regimen of ketamine also yielded no change both on firing rate (Figure 1(c)) and burst activity (33%) of 5-HT neurons, compared to two-day vehicle-administered animals (22%).

### Effects of acute and two-day administration of ketamine on the firing activity of VTA DA neurons

**Figure 2.**
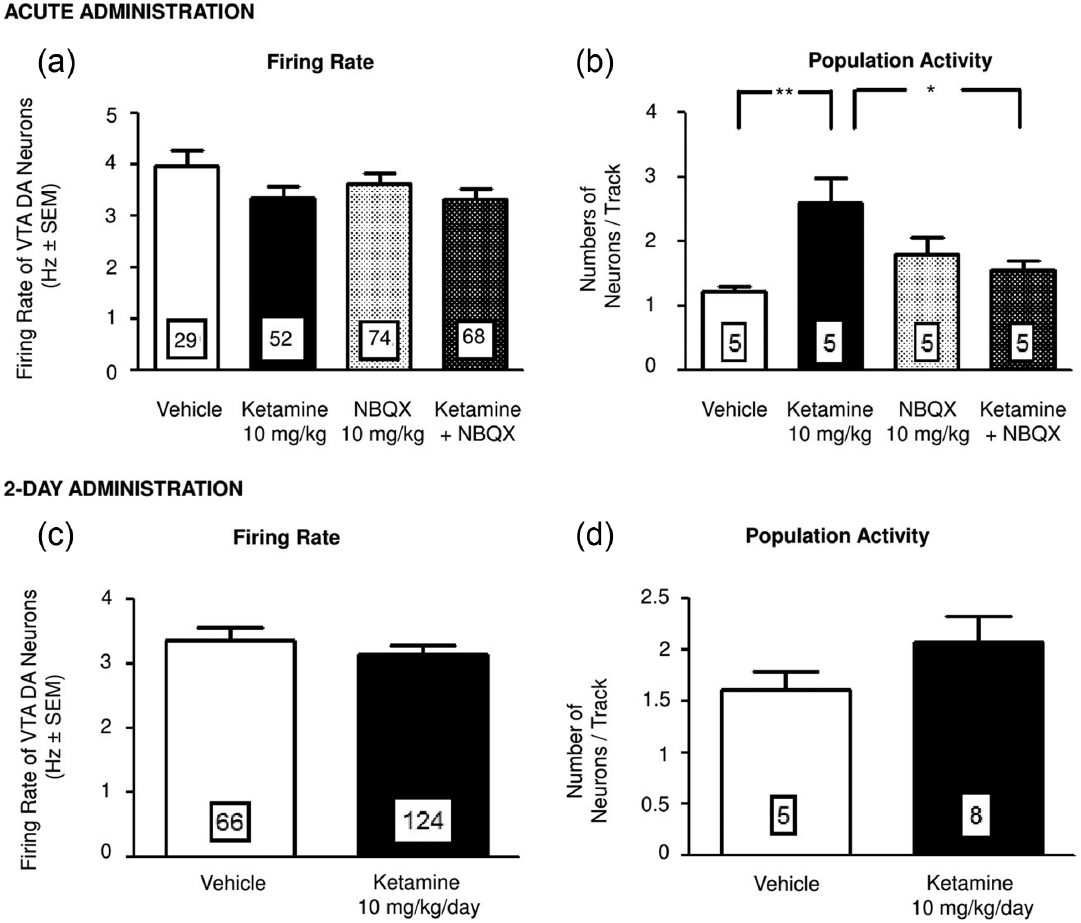
The effects of acute and two-day ketamine administration on VTA DA neuron firing. Mean (± SEM) of the firing rate and population activity of DA neurons following acute (a) and (b) and two-day (c) and (d) administration of vehicle or ketamine (10 mg/kg). The numbers in the histograms correspond to either the number of neurons recorded (a) and (c) or the number of rats tested per group (b) and (d). **p < 0.01; *p < 0.05.

After both acute and two-day administration of ketamine, the firing rate of DA neurons was unaltered compared to rats administered with vehicle (Figure 2(a), (c)). Moreover, no alteration in the burst activity of these neurons was observed (data not shown).

Despite an absence of effect of acute administration of ketamine on the rate of DA neuronal firing and bursting, the number of neurons encountered per electrode descent (population activity) increased by 113% (one-way ANOVA; *F*(3, 16)=5.4; *p*<0.01; Tukey post hoc test; *n*=5; Figure 2(b)) compared to vehicle-treated rats. The administration of the AMPA receptor antagonist NBQX (10 mg/kg) alone had no effect on DA neurons firing; in animals pretreated with NBQX, the increase of population activity after ketamine administration was no longer present (Figure 2(b)).

Similarly, following two-day ketamine regimen, the increase in population activity that was seen following its acute injection was no longer observed (Figure 2(d)).

### Effects of acute and two-day administration of ketamine on the firing activity of LC NE neurons

**Figure 3.**
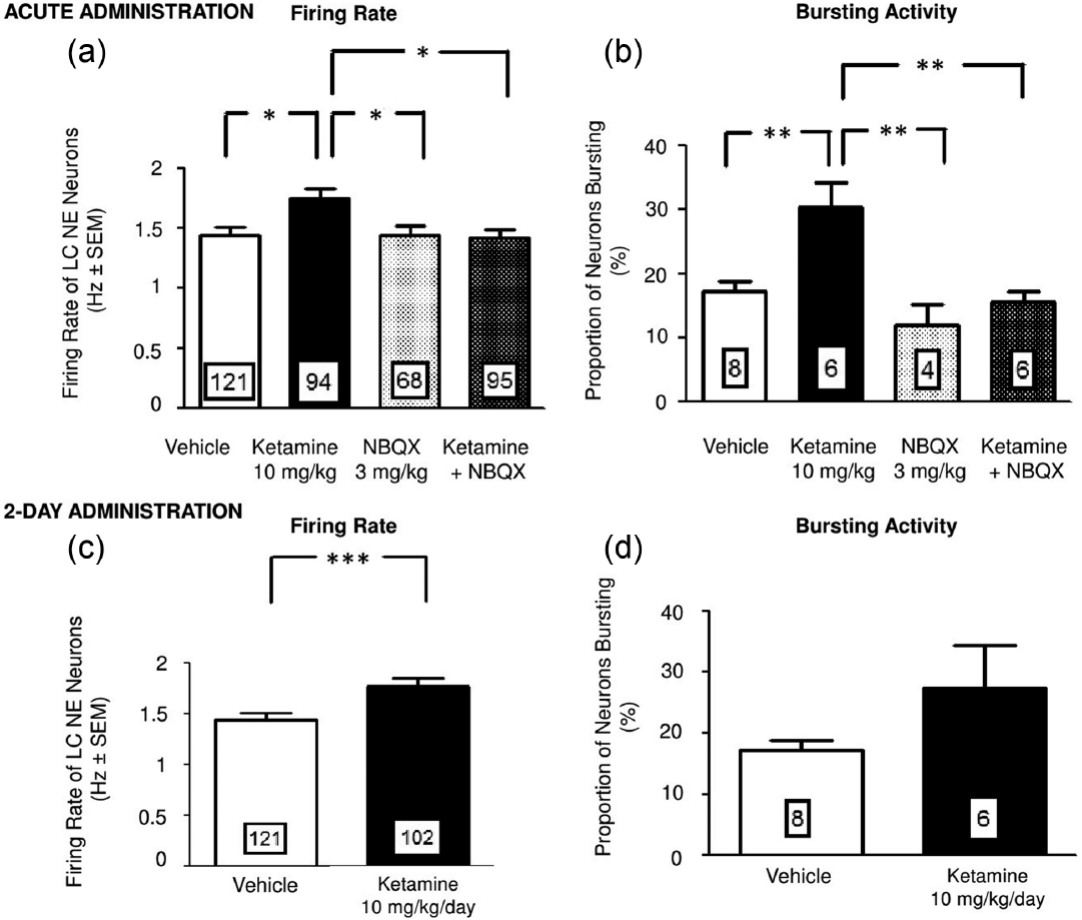
The effects of acute and two-day ketamine administration on LC NE neuron firing. Mean (± SEM) of the firing rate and burst activity of NE neurons following acute (a) and (b) and two-day (c) and (d) administration of vehicle or ketamine (10 mg/kg). The numbers in the histograms correspond to either the number of neurons recorded (a) and (c) or the number of rats tested per group (b) and (d). **p < 0.01; *p < 0.05.

Acute administration of ketamine resulted in a significant elevation of the average rate of firing activity of NE neurons in the LC by 21% when compared to the vehicle-administered group (one-way ANOVA; *F*(3, 375)=4.5; *p*<0.05; Tukey post hoc test; *n*=6; Figure 3(a)). However, this ketamine-induced increase in firing rate was not present in groups that were pretreated with NBQX (Figure 3(a)).

After a two-day administration regimen of ketamine, the enhancement in firing rate of NE neurons was significantly maintained to a similar degree (23%; Mann–Whitney Rank sum; *p*<0.01; *n*=6; Figure 3(c)).

In addition, acute administration of ketamine nearly doubled the proportion of NE neurons displaying burst activity (control: 17% versus ketamine: 30%; one-way ANOVA; *p*<0.01; *F*(3, 20)=9; Bonferroni post hoc test; *n*=6; Figure 3(b)). Since NBQX by itself had an effect on the NE neurons firing when injected at 10 mg/kg, an alternative dose of 3 mg/kg that has no effect was used. At this dose, pre-treatment with NBQX resulted in a dampening in the increase of the number of neurons with burst activity induced by acute ketamine administration (Figure 3(b)). Following a two-day administration regimen, the ketamine-induced increase in burst activity was no longer present (Figure 3(d)).

### Effects of acute ketamine administration on the responsiveness of hippocampus pyramidal neurons

**Figure 4.**
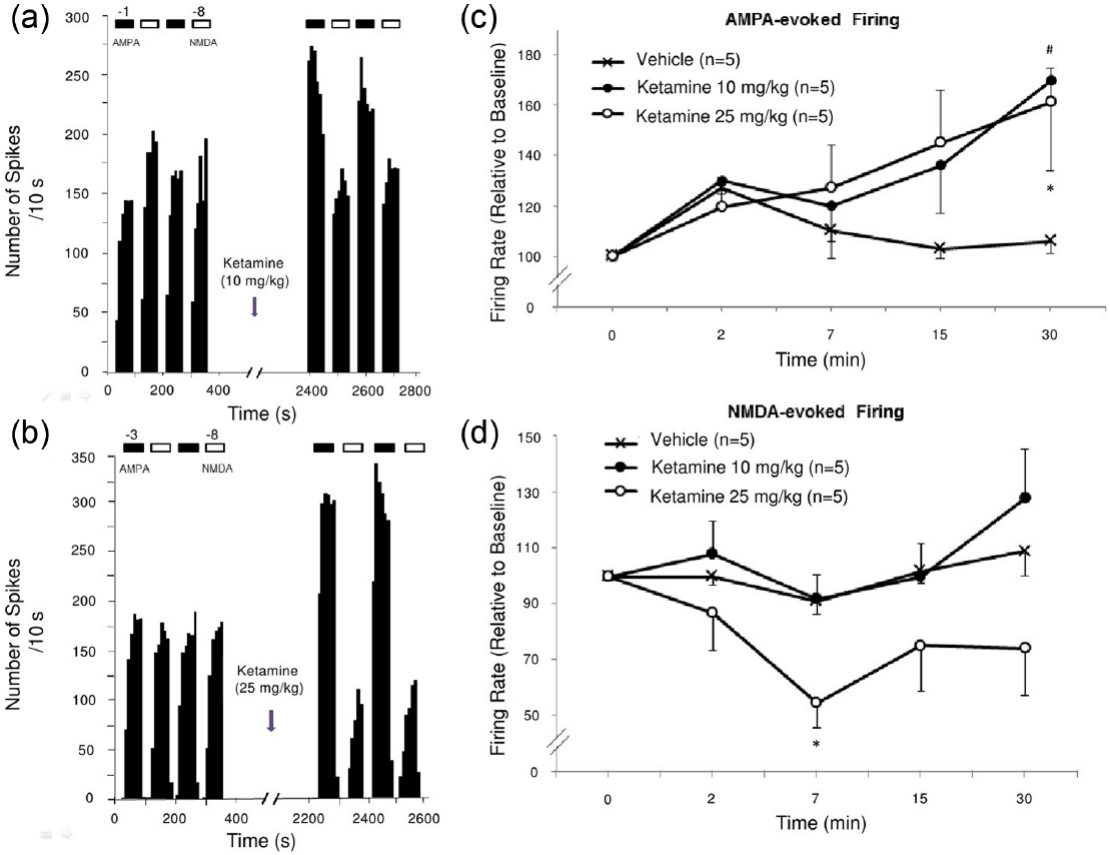
The effects of acute ketamine administration on the responsiveness of AMPA and NMDA receptors. Integrated firing rate histograms of dorsal hippocampus CA3 pyramidal neurons showing their responsiveness to ketamine administration (indicated by arrows) at a dose of 10 mg/kg (a), and 25 mg/kg (b). Horizontal bars indicate the duration of iontophoretic applications of AMPA (black) or NMDA (white). Ejection currents of -1 nA for AMPA and -8 nA for NMDA were used in this example. In (c) and (d), results are expressed as overall changes in the percent of baseline firing rate of dorsal hippocampus CA3 pyramidal neurons following administration of ketamine or vehicle; *p<0.05.

There was no significant overall main effect of ketamine (10 and 25 mg/kg) on the number of spikes generated per nA in response to iontophoretically-applied AMPA on pyramidal neurons. However, a significant time interaction was obtained 30 minutes following ketamine administration (using 10 or 25 mg/kg, showing a 64% increase in AMPA-induced firing activity of pyramidal neurons compared to control rat that received saline (two-way ANOVA with repeated measures followed by Bonferroni post hoc test; *F*(2, 48)=6; *P*<0.05; Figure 4(a), (c)). However, there was no alteration in responsiveness of these neurons to iontophoresed NMDA, as indicated by a lack of significant change in pyramidal neuron firing activity to iontophoretically-applied NMDA when rats receiving ketamine and vehicle were compared (Figure 4(a), (d)).

As a 10 mg/kg dose of ketamine did not change NMDA-induced firing activity of pyramidal neurons, a higher yet still subanesthetic dose was used (25 mg/kg; Figure 4(b)). As was the case with the lower dose, a 25 mg/kg dose of ketamine produced no overall effect on NMDA-evoked firing activity when compared to the vehicle treated group (Figure 4(b), (d)). However, a significant increase in AMPA-evoked firing was observed 30 minutes following the higher ketamine administration compared to control rats that received saline (55% increase; two-way ANOVA followed by Bonferroni post hoc test; *p*<0.05; Figure 4(b), (c)).

## Discussion

Several studies have already reported the involvement of the 5-HT system in the effect of ketamine. Indeed, ketamine administration reversed the 5-HT-induced reduction of excitatory postsynaptic current (EPSC) amplitude and frequency, observed in layer V of the prefrontal cortex after chronic unpredictable stress (Li et al., 2011). In addition, although the antidepressant-like effects of ketamine in the FST was elicited with 25 mg/kg but not 10 mg/kg (see Li et al., 2010), they were abolished following p-chlorophenylalanine administration that lowers 5-HT levels (Gigliucci et al., 2013). In the present study, however, the lack of effect on the firing activity of 5-HT neurons after both acute and two-day administration cannot be attributed to using an inadequate dose of ketamine, since a higher dose (25 mg/kg) was also without effect, whereas significant change were obtained on the firing activity of NE, DA and pyramidal neurons when using the low dose. The modulation exerted on 5-HT neuronal firing was previously shown to be through AMPA and NMDA receptors, since iontophoretic applications of AMPA and NMDA increased their firing activity that was blocked by glutamate receptor antagonists (Gartside et al., 2007). Consequently, the increase in glutamate release following NMDA blockade by ketamine (Lorrain et al., 2003a; Moghaddam et al., 1997) would have increased firing activity of 5-HT neurons through AMPA receptors. However, it was previously shown that the AMPA response was a direct effect on 5-HT neurons rather than resulting from local release of glutamate (Gartside et al., 2007). Therefore, since ketamine acts through the latter mechanism, this may explain its lack of effect on 5-HT neurons. Despite unchanged 5-HT neuronal activity, other studies showed that acute administration of ketamine (25 mg/kg) induced a transient increase in the 5-HT efflux in medial prefrontal cortex in awake rats (López-Gil et al., 2006; Lorrain et al., 2003b). In order to determine whether this effect was due to stimulation of 5-HT neurons or local action in the prefrontal cortex, c-Fos experiments were carried out (Lopez-Gil et al., 2006). These results revealed that the increase in the number of c-Fos-positive cells following administration of the NMDA antagonist MK-801 was altered by application of tetrodotoxin in the medial prefrontal cortex (mPFC), but not the in DRN. While this result indicates an involvement of the mPFC, it did not show a greater activation in DRN following NMDA receptor blockade (Lopez-Gil et al., 2011), which is concordant with results obtained herein on the firing activity of 5-HT neurons. Altogether, these data show that although there was no change in firing activity of 5-HT neurons, an increase of 5-HT neurotransmission can be observed in projection areas following acute ketamine administration. Future experiments will be undertaken to determine electrophysiological changes in 5-HT transmission in projection areas such as the hippocampus and the frontal cortex.

A previous study (French and Ceci, 1990) showed that i.v. injection of ketamine increased the firing rate of DA neurons with only a single neuron tested per rat. However, the present study showed no change in this rate in a sample of neurons recorded in several electrode tracks. The discrepancy may stem from the use of different methods (a single neuron versus a sample of neurons) in addition to the dose of ketamine used in the aforementioned study, which was twice the one used in the present experiments. However, the current study showed an enhancement in population activity of DA neurons, yielding possibly an increased response in the DA phasic bursts, therefore amplifying the salience signal (Lodge and Grace, 2006). This increase is concordant with the recently reported data showing that ketamine reversed a decrease in DA neurons population activity in Wistar–Kyoto rats exposed to inescapable and uncontrollable footshocks (Belujon and Grace, 2014). Furthermore, the present study also showed that the increase in population activity following ketamine in naïve rats was prevented by administration of the AMPA receptor antagonist NBQX, indicating that this effect is mediated by AMPA receptors. The involvement of AMPA receptors in the VTA is supported by data showing that local application of AMPA on DA neurons stimulates their neuronal firing (Tong et al., 1996; Zhang et al., 1997). Interestingly, it was also shown that a subanesthetic dose of ketamine increased the release of DA in the prefrontal cortex of conscious rats, through an action on AMPA receptors, because intra-PFC application of the AMPA receptor antagonist, 6-cyano-7-nitroquinoxaline-2,3-dione (CNQX), blocked this effect (Moghaddam et al., 1997).

This effect of ketamine on population activity of DA neurons coincides with previous studies revealing the major role glutamatergic afferents play in modulating DA population activity. Indeed, activation of glutamatergic afferents originating in the hippocampus stimulate the ventral subiculum, thus resulting in an inhibition of the GABAergic neurons of the ventral pallidum, thereby relieving its tonic inhibition of the VTA DA neurons (Floresco et al., 2001). Although the role of NMDA receptors was reported (Floresco et al., 2001), the present study showed an increase in AMPA receptor activation in the dorsal hippocampus following ketamine administration, providing a possible additional mechanism involved in the elevation of population activity in the VTA. However, following a two-day regimen, the ketamine-induced increase in population activity was no longer present, indicating that it is unlikely to contribute to the sustained effect of this drug. While an increase in DA population activity may alone be insufficient in mediating the antidepressant effects of ketamine, this enhancement may contribute to its immediate therapeutic effects, given the rapid increase resulting from acute administration, as was observed here.

Data from this study showed that acute administration of ketamine resulted in a significant increase in the firing rate of NE neurons and a doubling in the number of neurons exhibiting burst activity. Interestingly, when the LC was stimulated with burst pulses, it was previously shown that it enhances prefrontal cortex NE levels significantly higher than when tonic stimulation was applied (Florin-Lechner et al., 1996). Indeed, using microdialysis paradigm, Lorrain et al., (2003b) revealed that upon acute challenge, ketamine (12.5–50 mg/kg) increases hippocampal NE release that was blocked by the administration of AMPA/kainate receptor antagonist CNQX. This increased release is congruent with the enhancement in firing and burst activities of NE neurons observed in the present work. Furthermore, the latter was reversed by the selective AMPA receptor antagonist NBQX, indicating that this increased activity in LC is a AMPA-dependent effect. Interestingly, it was previously shown that the effect of glutamate upon NE neurons is largely of excitatory nature as demonstrated, for instance, by the fact that the AMPA antagonist LY293558 concentration-dependently blocked that effect (Rasmussen et al., 1996; see Jodo and Aston-Jones, 1997). The present result suggests that the increase in burst activity of NE neurons, which is mediated by AMPA receptors, might be contributory to the early antidepressant effects of ketamine. However, since the latter effect is not sustained, it may explain the lack of lasting antidepressant effects of this drug. Further studies are required in order to determine whether ketamine exerts a direct effect on LC NE neurons, or indirectly through the PFC or other structures.

In the present study ketamine administration resulted in an enhancement of AMPA- but not NMDA-evoked response in the hippocampus, suggesting that ketamine may exert a direct effect on AMPA receptors. Interestingly, chronic administration of ketamine resulted in a significant increase in AMPA but not NMDA receptor density in Wistar–Kyoto rats (a model for depression), whereas basal densities of these receptors were not significantly different when compared to Wistar rats (Tizabi et al., 2012). A synaptic potentiation of AMPA-mediated evoked neurotransmission was also shown in CA1 hippocampal slices. Furthermore, this was coupled with an increase in surface expression of both GluA1 and GluA2 subunits of AMPA receptors, which was inhibited by an AMPA receptor antagonist 6,7-dinitroquinoxaline-2,3-dione (DNQX; Nosyreva et al., 2013). In addition, following acute ketamine application, an enhancement in synaptic efficacy was also observed at rest condition (Autry et al., 2011). In the present study, however, the lack of blockade of NMDA-evoked firing of glutamatergic pyramidal neurons by ketamine may appear puzzling, but it could stem from the fact that ketamine exerts its effect through GABA interneurons. Indeed, blockade by MK-801 of NMDA receptors located on GABA neurons leads to decreased inhibition (disinhibition) following a surge in glutamate, thus resulting in an enhancement of AMPA activation (Abdallah et al., 2014; Homayoun and Moghaddam, 2007). Future studies on the effect of ketamine on GABA interneurons in the hippocampus are needed to confirm this issue.

The current experiments were carried out in rats that were under chloral hydrate anesthesia, which is known to affect glutamatergic transmission. Therefore, under such conditions the absolute changes produced by ketamine may be different from those occurring in conscious freely-moving rats. However, several studies were able to detect positive effects when measuring glutamatergic transmission under chloral hydrate anesthesia (see Christophersen and Meltzer, 1995; McCardle and Gartside, 2012). Moreover, since chloral hydrate inhibits different types of glutamatergic receptors, it is possible that effects studied under this anesthetic are rather underestimated.

In summary, ketamine administration resulted in an increase in catecholamine activity, and this increase was AMPA-dependent. It is noteworthy that the time-course of the effects of ketamine on AMPA receptors in the hippocampus was consistent with the time-course in which the increase in catecholaminergic firing activity began to occur. These effects could be a consequence of a direct effect of ketamine on NE and DA neurons, or indirectly due to an effect of ketamine on their glutamatergic afferents which have previously been shown to control monoaminergic neuron firing (Carlsson et al., 2001; Millan, 2006; Paul and Skolnick, 2003). Moreover, this study suggests that the antidepressant effect of ketamine could be occurring directly on the glutamatergic system, as shown by the increase in AMPA receptors responsiveness in the hippocampus (see Papp and Moryl, 1994). Whether the immediate effect of ketamine is dependent on glutamate, monoamines or their interaction remains to be elucidated.
